# Florid Cemento-Osseous Dysplasia with Superimposed Infection Mimicking MRONJ and Plasma Cell Neoplasia: A Clinicoradiopathologic Image-Based Challenge

**DOI:** 10.3390/diagnostics16121810

**Published:** 2026-06-11

**Authors:** Ömer Uranbey, Suat Aktaş, Kamil Nelke, Büşra Ekinci, India Maag, Filip Kulewicz

**Affiliations:** 1 Department of Oral and Maxillofacial Surgery, Faculty of Dentistry, Aydın Adnan Menderes University, Aydın 09100, Türkiye; suataktas@adu.edu.tr; 2 Maxillo-Facial Surgery Ward, EMC Hospital, Pilczycka 144, 54-144 Wrocław, Poland; kamil.nelke@gmail.com; 3 Academy of Applied Sciences, Health Department, Academy of Silesius in Wałbrzych, Zamkowa 4, 58-300 Wałbrzych, Poland; 4 Department of Medical Pathology, Faculty of Medicine, Aydın Adnan Menderes University, Aydın 09010, Türkiye; busra.ekinci@adu.edu.tr; 5 Private Dental Office, Volunteer Assistant, 54-144 Wrocław, Poland; 6 Aesthetic Medicine and Dental Implantology Specialised Practice, Śniadeckich 53, 51-604 Wrocław, Poland; wroclawimplant@gmail.com

**Keywords:** florid cemento-osseous dysplasia, MRONJ, multiple myeloma, secondary osteomyelitis, immunohistochemistry

## Abstract

Florid cemento-osseous dysplasia (FCOD) is a benign fibro-osseous condition typically presenting as multifocal, sclerotic masses throughout the jaws. While often asymptomatic, the hypovascular nature of the dysplastic bone predisposes it to secondary infection, which can mimic more aggressive pathologies. We present a complex diagnostic challenge involving a 76-year-old female with a history of intravenous ibandronic acid therapy for 18 months. The patient presented with a purulent mandibular fistula and exposed bone, leading to an initial clinical suspicion of Stage 2 Medication-Related Osteonecrosis of the Jaw (MRONJ). Radiographic evaluation, however, revealed generalized “cotton-wool” opacities across all four quadrants, characteristic of FCOD, with multifocal mixed radiolucent–radiopaque changes and a localized demarcated sequestrum in the symptomatic area. Accordingly, the diagnostic reasoning progressed from an initial clinical suspicion of MRONJ, to radiologic consideration of infected FCOD, and finally to exclusion of plasma cell neoplasia by immunohistochemical evaluation. The diagnostic dilemma intensified during histopathological analysis, which revealed an unusually dense, sheet-like infiltration of plasma cells within the fibro-osseous stroma. This striking plasmacytosis initially raised suspicion for a plasma cell neoplasm, such as plasmacytoma or multiple myeloma. To differentiate reactive inflammation from malignancy, immunohistochemical staining for kappa and lambda light chains was performed, demonstrating a polyclonal pattern that confirmed a reactive process. The final diagnosis was determined as FCOD with superimposed secondary osteomyelitis. Following conservative surgical debridement and targeted antibiotic therapy, the patient showed clinical resolution and remained stable at a 6-month follow-up. Recognizing these overlaps is essential for preventing over-treatment and ensuring appropriate management in elderly patients with complex medical backgrounds.

**Figure 1:**
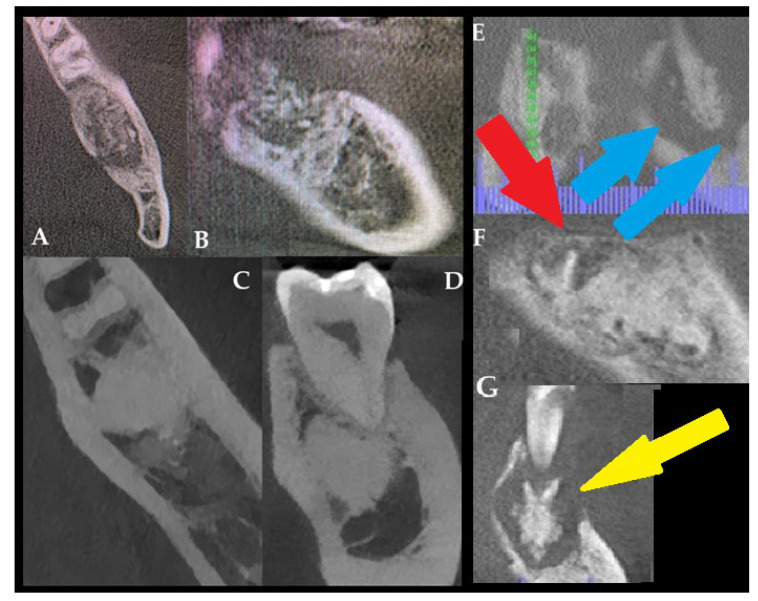
The scope of bone lesions that can manifest themselves in the mandibular body might include various radiological situations. In a healthy mandible, the outer surface is composed of cortical bone, while the internal structure consists of well-defined cancellous bone. The size, distribution, and density of trabecular bone may vary in appearance. Because of both mandibular bone anatomy, as well as some bone lesions that can be found in the mandible, the understanding of bone anatomy and radiological appearances is the most important factor helping in each lesion diagnosis and differentiation from other bone diseases. While fibrous, osseous, and fibro-osseous lesions are commonly encountered in the mandible, clinicians should also be aware that metabolic, endocrine, metastatic, and drug-related conditions may affect the mandibular bone. In these situations, some conditions may mimic each other [1]. In particular, Florid Cemento-Osseous Dysplasia (FCOD) may show a broad radiological spectrum depending on the stage, activity, and secondary changes of the lesion, ranging from radiolucent or mixed radiolucent–radiopaque areas to dense sclerotic masses. Therefore, its diagnosis is mainly based on the correlation of clinical history, lesion distribution, radiological maturation pattern, and exclusion of other fibro-osseous, inflammatory, metabolic, or neoplastic conditions. A close radiological differential diagnosis is quite easy; however, in some cases, patients’ medical anamneses about past surgeries, medication intake, and other general diseases present might influence the final diagnosis [2]. The problem arises where a biopsy is needed because of a possibility of the occurrence of a possible benign/malignant lesion [3] or co-occurrence of inflammatory factors, worrisome loss of inferior alveolar nerve function, extra-cortical spread, and other situations of lesions with irregular borders with both teeth and bone erosion [4]. On the other hand, developed local infection or primary or secondary origins might influence not only the bone structure but also the lesion within the bone, and then the diagnosis is more challenging [5]. The presented figure represents quite interesting mandibular bone structure changes associated with various clinical and radiological situations, namely (**A**,**B**)—inflammation and fibrous tissue formation along grafted allogeneic bone used to fill a defect after a dentigerous cyst removal (DC) with a mixed radiolucent and radiopaque appearance. On the other hand, (**C**,**D**) represents a DBI/IBO known as dense bone island or idiopathic bone osteosclerosis, where the lesion is radiopaque, well-defined without any osteolytic borders or a classic appearance of the most common fibro-osseous lesions like COD (cemento-osseous dysplasia) or its local or florid forms. Images (**E**–**G**) represent a rare situation of FCOD inflammation, formation with purulent fistulas, loss of bone, and extracortical spread, caused by poor dental status and lots of inflamed dental roots. COD/FCOD does not commonly become inflamed, but when present, the scope of cortical bone loss can have different shape, sizes and radiolucency (blue, red arrows) associated with granulation tissue and bacterial spread, in addition to notable extracortical spread (yellow arrow) when the inflammation extends outside of the cortices causing fistulas, mucosal swelling and pus secretion (green horizontal and vertical purple lines are orientation lines in the sectoral CBCT). These images also emphasize that FCOD does not always present with a single classic appearance; depending on secondary infection, dental status, cortical involvement, and chronic inflammatory changes, its radiological pattern may differ markedly between patients and even between different jaw regions in the same patient. The presented yellow, red, and blue arrows are orientation lines from CBCT (cone-beam computed tomography). Because of the following, the knowledge of the proper anatomy of mandibular bone and possible changes in its bony structure might be very useful to diagnose vast mandibular bone lesions (MBLs) [6].

**Figure 2:**
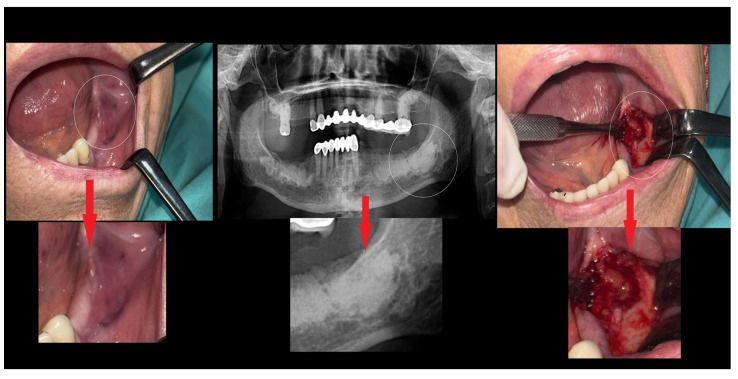
The diagnostic landscape of the mandible in elderly patients is frequently complicated by the coexistence of systemic comorbidities and long-term pharmacological therapies. Inflammatory lesions in this region may range from localized odontogenic infections to more complex conditions like Medication-Related Osteonecrosis of the Jaw (MRONJ) or infected fibro-osseous dysplasias. Specifically, Florid Cemento-Osseous Dysplasia (FCOD) represents a unique diagnostic challenge; although typically asymptomatic and discovered incidentally, the avascular and highly mineralized nature of the dysplastic tissue predisposes it to secondary infection following minor trauma or tooth extraction [1,2]. When FCOD becomes symptomatic, the clinical presentation characterized by purulent discharge, sequestration, and persistent pain often mirrors the clinical criteria for MRONJ, particularly in patients with a history of antiresorptive use [3,4,5]. In such instances, distinguishing between a primarily drug-induced necrosis and an infected intrinsic bone lesion is critical, as the management strategies may differ significantly. In the present patient, the previous intravenous ibandronate exposure was therefore considered clinically important because it reasonably supported the initial suspicion of MRONJ. However, this medication history was not interpreted as diagnostic by itself, and it was weighed against the generalized bilateral fibro-osseous radiographic pattern, the presence of asymptomatic FCOD-like lesions in other quadrants, and the subsequent histopathologic findings. Studies have shown that while MRONJ is primarily a pharmacological side effect, the “cotton-wool” patterns of FCOD provide a fertile ground for secondary osteomyelitis, creating a “great mimicker” effect in clinical practice [5,6]. In the present case, a 76-year-old female with a history of intravenous ibandronic acid administration (18 months) for osteoporosis was referred for evaluation of a persistent mandibular fistula and atypical severe facial pain. Intraoral evaluation revealed a mucosal defect with probe-accessible bone exposure and foul-smelling purulent drainage, a clinical scenario initially suggestive of MRONJ Stage 2 (A); white circle. At this stage, the diagnostic work-up was structured in a stepwise manner. First, MRONJ was considered the leading clinical diagnosis because the patient had a history of intravenous antiresorptive therapy, exposed mandibular bone, purulent discharge, and pain. Nevertheless, the antiresorptive history mainly influenced the early diagnostic pathway and the need for extreme clinical caution, rather than determining the final diagnosis. However, the clinical findings alone were not sufficient to explain the generalized jaw involvement. Second, infected FCOD was considered after radiographic assessment demonstrated multifocal bilateral fibro-osseous alterations extending beyond the symptomatic area, rather than a single localized drug-related necrotic defect. Third, because the subsequent biopsy showed an unexpectedly dense plasma cell-rich inflammatory infiltrate, plasma cell neoplasia, including plasmacytoma or multiple myeloma, was included in the differential diagnosis and required immunohistochemical exclusion. However, the multifocal nature of the underlying bone alterations, coupled with the unusual intensity of the inflammatory response, necessitated a deeper clinicoradiopathologic investigation to rule out aggressive systemic pathologies or hematological malignancies [7,8]. Accordingly, the final management was planned as conservative debridement and infection control rather than extensive MRONJ-type resection, because the overall clinicoradiopathologic picture favored FCOD with secondary osteomyelitis rather than primary medication-related osteonecrosis.

**Figure 3:**
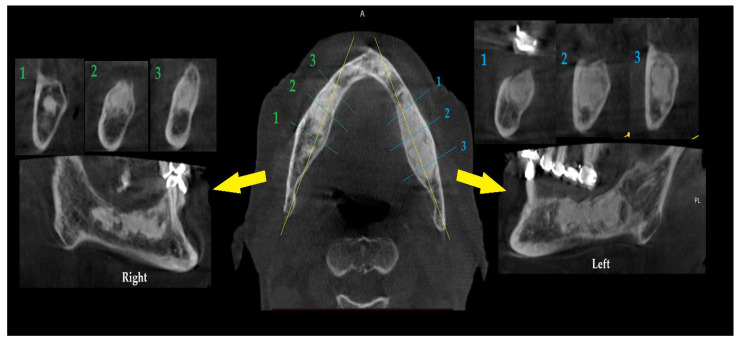
The panoramic radiograph revealed extensive, multifocal “cotton-wool” radiopaque lesions involving all four quadrants of the jaws, characteristic of Florid Cemento-Osseous Dysplasia (FCOD) with bilateral and multifocal distribution affecting both mandibular and maxillary tooth-bearing regions. The lesions showed mixed radiolucent–radiopaque internal architecture, with predominantly sclerotic mature areas and less mineralized radiolucent peripheral components, supporting a fibro-osseous maturation pattern rather than a purely destructive process. The yellow arrows indicate the longitudinal CBCT slice through the dental/alveolar crest, demonstrating a longitudinal view of the pathologic area. In the (**Left**) mandibular posterior region, the infected focus was clearly distinguished from the “silent” dysplastic areas by a distinct radiolucent demarcation line, indicating secondary osteomyelitis and sequestration. This symptomatic focus demonstrated a more irregular border than the remaining FCOD-related sclerotic masses, with a separated dense bone fragment compatible with sequestrum formation. The sequestrum was surrounded by a radiolucent rim, suggesting inflammatory separation from the adjacent dysplastic bone. The green and blue lines indicate the sagittal CBCT slices corresponding to the pathologic areas in the (**Right**, **Left**) mandible, respectively. CBCT provided a more detailed analysis, showing cortical expansion and the separation of the infected sequestrum from the surrounding dysplastic bone. CBCT also demonstrated focal cortical thinning and interruption in the symptomatic mandibular region, corresponding clinically to mucosal breakdown and exposed bone. In contrast, the non-infected FCOD areas maintained a more organized sclerotic pattern without the same degree of cortical disruption. These sagittal sections demonstrate that the fibro-osseous lesions originate within the medullary region of the mandible on both sides and may progressively extend toward the cortical bone over time. While the generalized radiopacities pointed toward FCOD, the localized bone destruction and sequestration closely mimicked the aggressive patterns seen in MRONJ or even chronic sclerosing osteomyelitis [9,10]. Therefore, the radiologic interpretation was not based on a single finding, but on the contrast between generalized, multifocal, relatively well-organized dysplastic bone changes and one localized symptomatic area showing demarcation, sequestration, cortical involvement, and secondary infection. This radiological duality complicates the clinical decision-making process, as dysplastic bone is highly avascular and prone to infection [11]. When cortical perforation and mucosal exposure occur, the dysplastic sclerotic bone may become exposed to the oral cavity, allowing for contamination by oral microorganisms. Given the poor vascularity of FCOD lesions, this exposure increases the risk of secondary infection, sequestration, and osteomyelitis [12,13]. Because the imaging findings still overlapped with MRONJ and chronic osteomyelitis, biopsy was performed from the symptomatic area to clarify whether the process represented infected FCOD alone or an additional neoplastic/inflammatory pathology.

**Figure 4:**
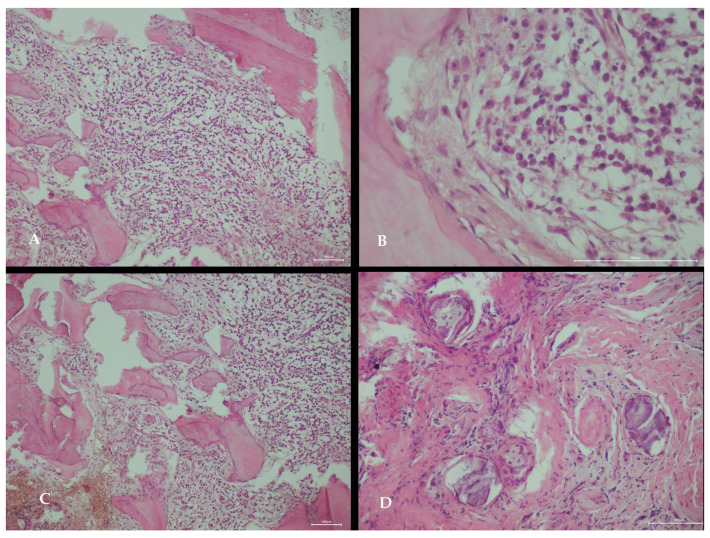
Histopathological evaluation of incisional biopsy specimens obtained from the limited area confirmed the fibro-osseous nature of the lesion. In the sections, dense, acellular, cement-like material was observed within the fibrovascular stroma, together with irregular woven bone trabeculae. The background consisted of hypocellular mineralized cemento-osseous tissue embedded in a chronically inflamed fibrovascular stroma, with irregular trabecular bone and cementum-like basophilic deposits showing variable maturation. In the symptomatic area, the mineralized tissue was accompanied by necrotic bone fragments, empty osteocytic lacunae, inflammatory granulation tissue, and mixed acute and chronic inflammatory cell infiltration, supporting secondary osteomyelitis/sequestration rather than a purely developmental fibro-osseous lesion. (**A**,**B**) H&E-stained sections demonstrated plasma cells completely filling the spaces between the bone trabeculae at both ×100 and ×400 magnification (BBA), highlighting the unusually extensive plasmacytic infiltrate. Notably, dense plasma cell infiltration was observed in the marrow-like spaces between the woven bone trabeculae. This infiltrate was not limited to a sparse perivascular distribution; instead, it formed broad sheet-like aggregates between the trabeculae and around the necrotic/inflamed fibro-osseous tissue, which was the main microscopic feature mimicking a plasma-cell neoplastic process. (**C**) At ×100 magnification (BBA), irregular woven bone trabeculae and dense plasma cells occupying the intervening stromal spaces were clearly visible, further illustrating the intimate admixture of fibro-osseous and inflammatory components. This dense cellularity was so prominent that it initially led to suspicion of a plasma cell neoplasm, such as plasmacytoma or a localized manifestation of multiple myeloma [14,15]. Thus, the differential diagnosis was further expanded at the histopathological stage: while the mineralized cementum-like material supported FCOD, the sheet-like plasma cell infiltrate required exclusion of monoclonal plasma cell proliferation. (**D**) Moreover, mineralized cementum-like particles embedded within the fibrous stroma were identified on H&E sections at ×200 magnification (BBA), supporting the diagnosis of a cemento-osseous lesion. The coexistence of cementum-like mineralized particles, irregular woven bone, necrotic/sequestrum-like bone, and plasma-cell-rich chronic inflammation supported the interpretation of infected FCOD with secondary osteomyelitis. In most fibro-osseous lesions, a moderate inflammatory infiltrate with mixed inflammatory cells, including neutrophils, histiocytes, and lymphocytes, is expected; however, in the present case, superimposed infection triggered an exaggerated reactive plasmacytosis, creating a significant diagnostic pitfall for the pathologist [16,17].

**Figure 5:**
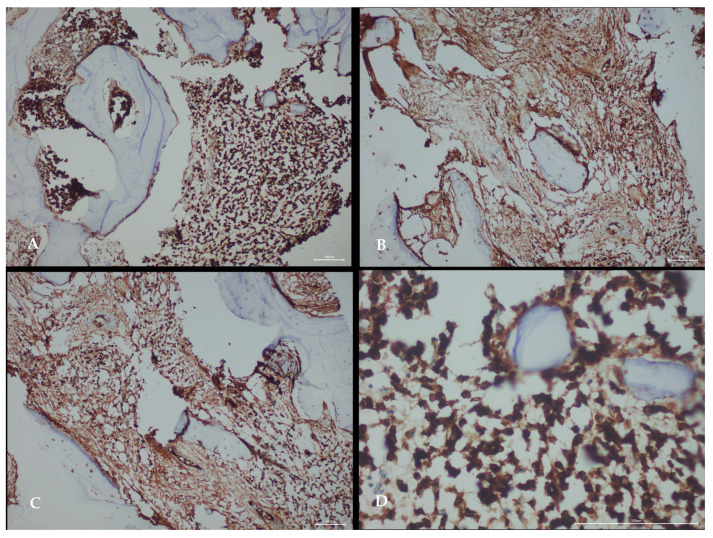
To resolve the diagnostic dilemma between reactive inflammation and malignancy, immunohistochemical (IHC) analysis was prioritized. (**A**) An additional CD38 immunostain demonstrated diffuse positive staining in plasma cells at ×100 magnification (BBA), further confirming the abundance and distribution of the plasmacytic infiltrate within the lesion. CD38 was interpreted as a plasma-cell marker and was used to confirm that the dense cellular component observed on H&E sections represented a true plasma-cell-rich infiltrate rather than nonspecific chronic inflammatory cellularity. This finding supported the interpretation that the striking cellular component seen on routine histopathology truly represented a plasma cell-dominant infiltrate and justified further characterization with light-chain analysis. (**B**,**C**) The IHC staining for kappa and lambda light chains at ×100 magnification (BBA) demonstrated a polyclonal expression pattern within the plasma cell population. The interpretation was based on the absence of light-chain restriction: both kappa-positive and lambda-positive plasma cells were present within the infiltrate, without exclusive predominance of a single light chain. This preserved dual light-chain expression supported a reactive/plasmacytic inflammatory response, whereas a marked predominance of only one light chain would have favored monoclonal plasma cell proliferation. The balanced staining of both light chains confirmed the reactive nature of the infiltrate, effectively excluding monoclonal plasma cell dyscrasias like multiple myeloma [18]. Following this confirmation, the pathology department concurred with the diagnosis of FCOD-related secondary osteomyelitis. (**D**) At higher magnification, diffuse CD38 positivity in plasma cells was again evident on immunohistochemistry (×400, BBA), further supporting the marked plasma cell-rich but reactive character of the lesion. Therefore, CD38 established the extent of plasmacytic infiltration, whereas kappa/lambda staining provided the decisive clonality assessment. The lack of kappa or lambda restriction argued against plasmacytoma or plasma cell myeloma and favored infection-driven reactive plasmacytosis in a dysplastic, necrotic cemento-osseous background. This finding underscores the necessity of IHC in cases where chronic osteomyelitis in dysplastic bone mimics hematological malignancies [19,20]. Thus, while CD38 highlighted the density of the plasma cell component, the kappa/lambda profile provided the decisive evidence that the process was reactive rather than neoplastic. This completed the stepwise diagnostic pathway by excluding plasma cell neoplasia and supporting the final diagnosis of FCOD with superimposed secondary osteomyelitis rather than primary MRONJ or hematologic malignancy.

**Figure 6:**
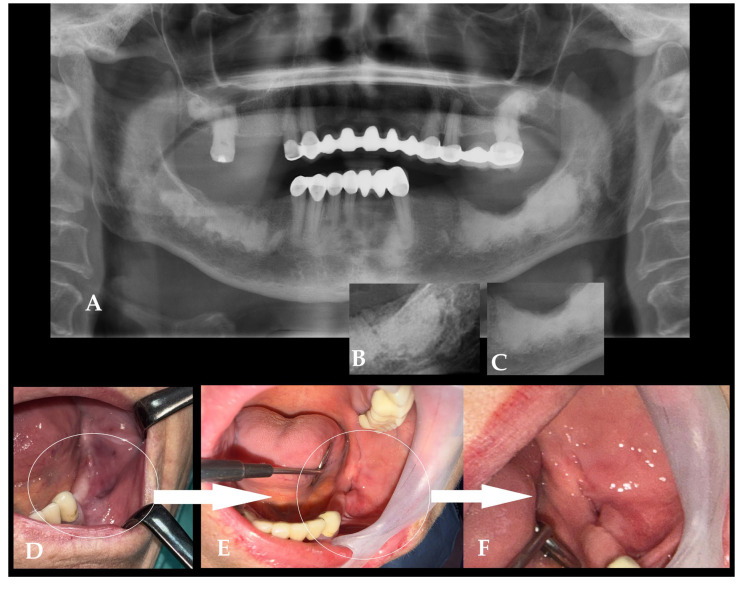
Following conservative surgical debridement and antibiotic therapy, the patient showed significant clinical improvement. A 6-month follow-up panoramic radiograph (OPG) demonstrates the stability of the FCOD lesions across all quadrants, with successful remodeling and no signs of recurrent infection or new fistula formation in the previously symptomatic site. (**A**) At the 5-month postoperative follow-up, the OPG demonstrates bone resolution and radiographic stability in the affected area. (**B**) In the magnified preoperative OPG view, the infected bone region appears with a clear demarcation line, whereas at the 5-month postoperative radiographic follow-up (**C**), the non-infected FCOD bone appears stabilized, with disappearance of the previously visible demarcation line. Unlike MRONJ, which often requires aggressive resection in advanced stages, this case illustrates that secondary infection in FCOD can be managed successfully through a combination of meticulous diagnostic workup and conservative intervention [21,22]. (**D**) Clinically, the fistula-associated intraoral gingival tissue represents the infected soft-tissue condition before complete healing. (**E**) At the 5-month postoperative intraoral follow-up, the gingival tissue demonstrates a non-infected and healed mucosal appearance. (**F**) The closer intraoral view further highlights the absence of fistula formation and the improved soft-tissue condition. Long-term monitoring remains essential, as the hypovascular nature of the remaining FCOD foci poses a lifelong risk for further infectious episodes [23]. Therefore, the 6-month follow-up in this case should be regarded as an early, favorable clinical outcome rather than definitive long-term disease control. This relatively short follow-up period represents a limitation of the present image-based case report, particularly because recurrent infection may develop later in untreated or residual FCOD areas. From the clinical and radiological perspective, the key learning points include the following: (1) cortical bone changes in advanced fibro-osseous lesions should be carefully differentiated from true inflammatory cortical de-struction and extracortical spread; (2) most COD/FCOD lesions remain asymptomatic, but close proximity to tooth apices should be monitored to exclude secondary odontogenic inflammation; (3) IBO/DBI usually presents with well-defined radiopaque borders and should be differentiated from osteoma, exostosis, tori, and fibro-osseous le-sions; (4) previous bone grafting or xenograft/allograft use may create radiopaque appearances that resemble IBO, COD, FCOD, or other fibro-osseous conditions, making patient history essential; (5) exposed grafted or autogenous bone may become contaminated by saliva and oral bacteria, leading to local inflammation and granulation tissue formation; (6) irregular radiolucency without clear borders, periosteal reaction, cortical destruction, or extracortical extension should prompt biopsy to exclude bone tumors or malignancy; (7) in patients with antiresorptive exposure, MRONJ should be considered, but medication history alone should not override a bilateral or multifocal COD/FCOD-like radiological pattern; (8) the distinction between generalized stable dysplastic bone and one local-ized symptomatic area with sequestration, demarcation, cortical perforation, or fistula formation is critical for di-agnosis; (9) biopsy should be directed to the symptomatic or radiologically atypical area when infection, malignan-cy, or hematologic disease is suspected; (10) dense plasma-cell-rich inflammation may mimic plasma cell neoplasia, and kappa/lambda light-chain assessment is useful for confirming a reactive rather than monoclonal process; and (11) conservative debridement and infection control may be appropriate in selected infected FCOD cases, although long-term follow-up remains necessary because residual dysplastic bone may become infected later.

## Data Availability

The raw data supporting the conclusions of this article will be made available by the authors on request.
